# Hospital and Institutional News

**Published:** 1919-04-26

**Authors:** 


### HOSPITAL AND INSTITUTIONAL NEWS.

### CANNES CONFERENCE AND TUBERCULOSIS.

The report on tuberculosis adopted by the Allied Red Cross Societies at the Cannes Conference runs on well-worn lines—the dispensary, inspection of school children, isolation hospitals for advanced cases, the sanatorium and popular propaganda. One looks in vain for any mention of the fact that in districts where all these measures had been in working order for some years, war privation almost instantly made the disease flare up into a great blaze in spite of all that could be done. In view of this phenomenon, which has naturally been much remarked on by responsible lay persons, it seems strange that Sir Robert Philip and his colleagues blinked all its implications. One of the chief of these is that the practical effect of the infection factor in tuberculosis has been much overrated, and that individual constitutional resistance is an anti-tuberculosis agency of hitherto vastly underrated significance. The best way to promote a good constitution in the proletariat is by general social amelioration. More than ever do we see that tuberculosis is almost more of a sociological problem than of a medical one.

### THE RABIES OUTBREAKS.

In spite of the very frequent reports that more mad dogs have been killed in various parts of the country, it would appear that this outbreak is far less serious than reading of the daily press would suggest. Very few of the recent instances are being confirmed as rabies by post-mortem examination of the animals. In any case the prevalence of the infection in England has not come upon the Board of Agriculture as a surprise, preparation has been made for the last eight or twelve months and people, if bitten, will find treatment according to Pasteur's methods available here in England. Undoubtedly the introduction comes from France or Belgium. In these countries the disease has long been prevalent and therefore the bringing home of dogs by our returning soldiers was strictly forbidden. Posters were set up in the war areas explaining to men the possible danger of the situation, but apparently love for the dog has been too strong, and in spite of detection and punishment in some cases, smuggling of the animals must on occasion have been successful.

### THE NATIVE HOSPITALS IN EGYPT.

In view of the disturbances in Egypt and the allegations in regard to the forced recruitment of labour battalions there, the following statement about the native hospitals which Mr. F. E. Wynne has included in his "Personal Experiences of the Egyptian Labour Corps" is worth reproducing from the *Manchester Guardian*. After attributing most of the difficulties to the venality of the Egyptian officers of the corps, who subsist, after their kind in Egypt, on *backsheesh*, Mr. Wynne says: "The native hospitals that I knew—and I knew many of them well at the base, on the lines of communication, and just behind the front—were well provided with tents and equipment, and there was always an English M.O. in charge. So far from being pest-houses, which people feared to approach, I remember one which was established within 100 yards of the casualty clearing-station to which I was for a time attached. The M.O. in charge of it was a member of our mess, and a man who would not have tolerated or permitted anything like the state of affairs I have seen suggested as being the rule, and I had no reason to regard this hospital as exceptional." The whole article is so interesting, and seems so judicious and well informed, that we commend it to our readers, who will find it in the issue of April 12.

### WOUND SHOCK.

The main points considered at the B.M.A. discussion of this section centred round the war division into (1) "primary shock," as usually seen in the front line, and probably due to a weakening of vasomotor tone; and (2) "secondary shock," seen in the clearing stations and stages of transport to the base. The opening papers and comments, of which we give a *précis* on a later page, covered a considerable amount of ground without being very definite in any particular quarter. The most prominent part of the proceedings was, perhaps, a comparison between blood transfusion and gum saline infusion. Undoubtedly the latter is a great improvement on previous saline methods, but one believes that at present practical experience definitely establishes the advantage of blood transfusion. Such, at least, is the opinion expressed by the majority of medical officers who have experience of both methods.

### METROPOLITAN ASYLUMS BOARD QUALIFICATION.

Dr. Addison, President of the Local Government Board, has notified the London boards of guardians that it has been decided to withdraw Article III of the Order made by the Poor Law Board 1867, which had been applied to the qualification of a nominated manager of the Metropolitan Asylums Board. This article stipulated that the manager should be assessed to the poor rate upon a net annual value of not less than £40 a year. Dr. Addison adds that it appears that this regulation might limit the field of selection open to the boards of guardians in electing their representatives on the Metropolitan Asylums Board. He prescribes that in future the qualification of an elective manager shall be determined solely by section 10 of the Act of 1867, which means that any person will now be eligible for election who is (*a*) a guardian of the electing union, or (*b*) a ratepayer qualified to be such a guardian. In view of the large number of Labour representatives in London recently elected to the various boards of guardians, the abolition of the property qualification for membership of the Metropolitan Asylums Board comes at an opportune time.

### THE MANIA FOR ORDERS.

In spite of the flood of decorations which is threatening to give all distinction to the plain man, the appetite seems to grow: a man without a medal is becoming a man of mark, and a woman without a decoration as a girl without her ornaments. The very brassards are prized. Deluged with demands for them as keepsakes, it has been decided that they may be kept, but, with official subtlety, that they must not be worn and that the War Office stamp must be removed from them. An ornament that cannot be worn is not very attractive to most women, who very naturally prefer to deck themselves. But is the mania for orders a proof of our democracy, or is it that we prefer "decorations" to distinction, the mark of which is simplicity and quiet?

### THEIR SECOND HOME.

A new type of institution is coming into being with the hostels now springing up for limbless and disabled men. In London five hostels (73-78 Queen's Gate, South Kensington) have now been opened by the Princess Royal. The same process is general. At Swansea an annexe to the hospital shows the link by which the separate hostels are connected with hospitals. The closing of the auxiliary hospitals has brought these hostels into being, which Mr. Roger Beck, at Swansea, hopes will become a second home to the men. The hostels, in so much as they prolong war hospitals into peace-time institutions, will, we hope, become permanent, and take their place in that reconstruction of our hospital system which is now beginning, and must be made complete by voluntary efforts if we are to escape a Germanised regimentation of our lives.

### THE TWO CONCEPTIONS OF VICTORY.

When Sir Robert Borden lately unveiled the "Victory" statue designed by the Australian artist, Mr. Bertram McKennell, at Cliveden, to the memory of the Canadian officers and men who had died at the Duchess of Connaught's Red Cross Hospital at Taplow, and who are buried at Cliveden, he pointed out that the memorial was as much to the devotion and self-sacrifice which had made victory possible, as to Victory itself. Now that memorials with an artistic claim are beginning to arise in different parts of the country, a word is in season on the contrasts which they offer to the classic treatment of the old theme, Victory. No one who has seen the winged Niké in the Louvre can doubt that it is the finest embodiment of the idea of Victory in the world. The height of the great statue, the sweep of its draperies as in a great wind, combine at once the stride of triumph with the suggestion of a dance. It is, as the idea of Victory should be, one of pure joy and health. Pathos is as far removed from it as the taint of mere success, or the vulgar crow of assertion. How different are the figures to which we have become accustomed, those anæmic and academic maidens with their blank faces, and timidly correct air, posturing in the void like an *ingenue* at her first ball! It is the pathos of suffering, rather than delight at the end which suffering has won, which seems to move the modern mind. Those who regard this change as an improvement are entitled to their opinion: but is not the old idea more congenial to the happy and joyous atmosphere of a hospital, if only one had the artist to give plastic form to it?

### THE SERVICES OF MR. ORDE.

We regret to have noticed that an attack has been made by certain working-class delegates upon the salary paid, among that of others, to Mr. Roden Orde, the distinguished secretary of Newcastle Royal Infirmary. The trouble is that the men who boast of the power of the penny in hospital finance in industrial districts are apt to think in pennies, and ask themselves not what type of man they want and the work requires, but what is the least that any one at all eligible can be induced to accept. They also are apt to measure ability by the time needed to get through the work to be done, and since as trades unionists they encourage the limitation of output, have little, if any, inkling of the standard set by the universities, which is that until a man has given his best he is a bad workman. Now the test of mastery is the ease with which a difficulty is mastered and a task completed; just as the test of drudgery is waste of time, which no skill can shorten. Since Mr. Orde's record could not be impugned except in so far that he had leisure to spend on other public work, should not his critics ask themselves what it is that they are paying for? As Mr. Orde is too modest to tell them, we will. They are paying him for his ability, his education, and his experience, and his remuneration is to be measured not by the hours he takes to do his work, but by the result accomplished; and that result is simply the fruit of the experience of a lifetime.

### THE COTTAGE HOSPITAL SECRETARY.

Cottage-hospital secretaries pursue the noiseless tenor of their way, and were largely unnoticed in their own country until an American writer not long ago held them and their institutions up to the admiration of Americans, urging them to do likewise. We are glad to see, therefore, that due expression has been given to the gratitude felt towards Mr. Evan Pugh, who for twelve years has been secretary to Rhymney Cottage Hospital in Wales. His recent, perhaps hasty, resignation has not been accepted. He has been unanimously re-elected, and should be proud enough of his record to surpass it, if he can.

### THE LATE DR. A. G. BATEMAN.

By the death after a short illness of Dr. A. G. Bateman, not only does the Medical Defence Union lose one of its founders and a Secretary of very marked personality, but the whole medical profession is poorer by one whose labours tended to benefit every registered man or woman. What Dr. Bateman was to the Defence Union, its Council, and those of its members who have had to seek his services know full well: suffice it on this head to remark on the difficulty that must inevitably be found in filling his place. To his fellow-practitioners at large Dr. Bateman was also a general benefactor, in that he diligently pursued the task of suppressing quackery and imposture as far as the very defective statutes dealing with medical practice in this country allow. He took a positive delight in detecting and prosecuting the impersonators of qualified practitioners and other forms of illegal practice; and it was largely due to his fervour in the quest that the Defence Union has for some years taken upon itself the burden of bringing these ruffians to justice. Dr. Bateman's collection of dossiers about the more notorious offenders were full of curious and valuable information: it is to be hoped they may be made available for the use of his successor in office.

### A CHAIRMAN'S RESIGNATION.

We are sorry to learn that Mr. C. C. Sibthorpe has been compelled through ill-health to resign the chairmanship of Lincoln County Hospital. He will probably retain the post of treasurer till the end of the financial year. It is five years since Mr. Sibthorpe succeeded Canon Hutton in the senior post, and they have been years of great anxiety and change, met with resource and energy. Mr. A. Moore, the superintendent, could testify to the value of his work.

### THE PARABLE OF PETERBOROUGH.

The Peterborough scheme for a new infirmary as a war memorial is "petering" out in apathy and indifference. We, and the people of the place, have this on the authority of the Mayor, who reminds them that the commonest excuse for their attitude is: "The Minister of Health will give us a new hospital and the State will maintain it." It is odd that the people of Peterborough should be so anxious to super-tax themselves, or can it be that they fail to realise that the "State" means simply the people who pay taxes? Unless they all belong to the leisured world of labour, the only world to-day which is free from direct taxation, they will soon awaken to the fact that they are adding to their burdens. But when that time comes they will also discover that it is easier to invoke "State aid" than to escape from its intolerable interference. The one man, apart from the Mayor, who keeps his head in Peterborough is Dr. Collins, the single man to accept the challenge provided by the donor of 1,000 guineas for the new hospital, of the nine on whose appearance the gift depended.

### A FINANCIAL POLICY FOR LIVERPOOL.

The Liverpool Council of Voluntary Aid includes among its services to the hospitals of the city an annual financial statement prepared, in the first instance, for the medical charities committee of the Council. The chief point shown this year is the new trend of hospital finance downwards. We say new, because the Council has observed that up to 1915 the total incomes of the Liverpool hospitals had exceeded their total expenditure. In 1916 the balance first appeared on the wrong side. In 1917 it was more marked still. In 1918 the situation is expected to have been worse. In short, a financial set-back has interrupted a strong financial tendency; and now that the revenue from military patients is fast coming to an end, while expenditure at least will be maintained, a true crux is reached. It is not the immediate business of the fund, or of its keen secretary, Mr. F. G. D'Aeth, to suggest a remedy. A cut-and-dried one cannot be found. Our hope now, as in the past, resides in the elasticity of the voluntary system. Therefore, appeals, severally or jointly, must be issued on the Birmingham model to extend or rebuild the hospitals so as to remedy the existing shortage of beds; those occupied by soldiers on the bungalow plan must be filled with civilians, and since the temporary structures have proved their worth, the money raised need not be wholly sunk in expensive bricks and mortar. Local authorities and public authorities who will claim many of these beds must by their payments help to replace the loss of "military revenue"; sectional, and large annual subscriptions (on the Cardiff plan) must be cultivated, and the organisation of war funds must be kept going for the benefit of the civil patients in the hospitals.

### THE BIRMINGHAM HOSPITAL COUNCIL.

Mr. Neville Chamberlain, M.P., is now supporting the local movement for co-ordinating the voluntary hospitals of the city so as to find a middle course between the unsolved alternatives of "bankruptcy" and State control. He urges the Lord Mayor's Hospital Council to bring the seventeen hospitals into closer touch, the General Hospital to give the lead, and the city to begin at the periphery the co-ordination which is about to come also from Whitehall. We should not criticise his suggestion were it not that Mr. Chamberlain declares that the hospitals must be prepared to sacrifice some independence. But the best argument for his plan really is that if the city hospitals combine in time they may be able to forestall and check the encroachments of Whitehall to their own and to their patients' advantage. The Lord Mayor and all interested should study "The Hospital City," see the *Encyclopædia Britannica*, 11th edition, volume xiii., pp. 795, 796.

### THE STIRRING OF BLACKPOOL.

The Victoria Hospital, Blackpool, whose history a few years ago was as sombre as the name of its locality, seems to be stirring into active life. It is like an echo of the old controversy to learn that "the completion of the new trust deed is now well in hand." But it is more cheering to learn also of the plans for the hospital's extension. Support has been promised, confidence is being restored, and we await details of the new schemes as soon as the sub-committee in charge of it shall have reported.

### THE BAD SAMARITAN.

We regret to notice in such Hospital Sunday reports as have yet reached us, far too little emphasis upon a point which will be new to most of those who listen to its appeal. We mean the way in which the scandal of the waiting-lists is kept in the background. Yet the man on the waiting-list is in precisely the same plight as the man who was rescued by the Good Samaritan, and the public should be told that they are, and remain, Bad Samaritans so long as there is no bed available for the sick.

### EISTEDDFOD GRANTS TO HOSPITALS.

Hospitals and nursing associations in and about the localities in which the Welsh National Eisteddfod is held year by year should take a more than passing interest in the financial success of the great festival in view of the manner in which the surplus funds are divided. There was a substantial profit on the Neath Eistoddfod last year, and among the grants which have been made therefrom are the following: £1,000 towards the erection of a general hospital at Neath, conditional upon it being erected within five years, the interest on the capital meanwhile to be given to the Swansea General Hospital; £100 to Neath Nursing Association; £50 each to Aberavon and District Hospital and the National Institution for the Blind; £25 to Swansea Hospital; £20 each to the Aberavon, Skewen and Briton Ferry Nursing Associations; and £10 to the Bryncoch Nursing Association.

### THE WOMAN COMMITTEE-MAN OBJECTED TO.

By an innovation it has been decided to add to the board of Leeds General Infirmary two seats to be occupied by women. Medical men joined in stating that women would strengthen the board because on some points their advice was specially valuable. A lay member however said, truly enough, that there were "places a woman could not fill," and thought the place of a committee-man was one of them. In fact, the speaker, Mr. George Brown, refused to serve on a mixed board. The "emancipation" of women was bound to produce a reaction.

### PEACE HAS HER CASUALTIES.

It was a pleasure to notice that the local Health Committee persuaded Miss Laing to withdraw her resignation of the matronship of the Moss Lane Hospital, Southport. After twenty-six years' work, Miss Laing was entitled to consideration, especially since her breakdown in health was attributed to war work. It was, therefore, graceful to offer three months' leave of absence. We hope the rest will renew her strength and lead her back to hospital work again.

### AN EXAMPLE OF HOSPITAL PRINTING.

The first number of the *Netley Red Cross Hospital Magazine* (Vol. II.) is a thoroughly home-made product, for it comes fresh from the hospital's own press, situated in the orthopædic workshops. The new number marks the transition of the hospital from an institution for wounded to an (shall we say?) armistice institution for disabled and other men. Edited by Mr. Cæsar Caine, the new number is extra-illustrated with a drawing by Mr. W. Heath Robinson, called "The Hairoplane," reputed to comb and brush American scalps, which are seen emerging like cabbages from a wonderful neat trench. The workshops will long remain a memorial of Red Cross endeavour, and the details of the work there being done compensate for the disappearance of the Welsh Hospital on March 21. The magazine at present is richer in illustrations than in articles, in jests than in information, in verse than in prose. But its printers, under Captain Hylton Lamotte, are the heroes of the new issue, and if the editor be as well served by his contributors as he is already by his compositors, it will become a first-rate publication. If the readers in the hospital follow the standard set for them by the "reader" in the workshop, all will be well. The printers have given a lead. It is for the readers to supply them with good "copy."

### THE CURE FOR ARMY BOREDOM.

The Birmingham and District Professions and Trades Fund for the entertainment of wounded sailors and soldiers has often been mentioned in our columns for the vast series of concerts and amusements which it has successfully arranged, not in one hospital, but in many. An expression of thanks has now been received from the Headquarters of the Southern Command for "the wonderful work" accomplished. This shows that the authorities benefit no less than the men by the provision of entertainments for the latter. Army work, except on active service, is so like housemaid's work that the worst enemy of the soldier is the boredom of his life. To mitigate this civilian help had to come to the rescue, and without it the Army and the wounded would have remained in a sorry plight indeed.

### PUERICULTURE IN NOTTINGHAM

A recent number of the *Nottingham Journal* contained an interesting account of the county public health work amongst children. In Nottingham open-air classes for delicate children have been established in centres distributed over the city, from Bulwell Forest to the Notts Football Ground. Six clinics are also at work, one for dental treatment, the yearly attendances at all reaching as high a number as 100,000. Ophthalmic treatment, including glasses at cost price, $x$-ray treatment for ringworm, scrofula and lupus, massage, surgical appliances, are also all obtainable at these places.

### A POSTHUMOUS CRITICISM.

Personalities in wills have been criticised adversely by judges before now; but what is to be said of a criticism directed at a hospital from the safe corner of a codicil? To the lately published will of Mr. William Davidson, an Inverness merchant, was attached the following note: "Under a previous settlement (now cancelled by me) I bequeathed a legacy of £300, afterwards increased to £500, to the Bignold Cottage Hospital, Wick, and since then I have reluctantly been compelled to the conclusion that the said hospital is not managed or administered as it should be in the best interests of the patients, but rather in a personal and political sense, and not at all in keeping with or calculated to exert that impartial power for good which such an institution run on neutral and purely humanitarian principles would bring about. I leave it to the discretion of my trustees to pay or withhold the said sum." Might he not have left this statement to their discretion also?

